# Lupeol-3-carbamate Derivatives: Synthesis and Biological Evaluation as Potential Antitumor Agents

**DOI:** 10.3390/molecules29173990

**Published:** 2024-08-23

**Authors:** Shuang Tian, Yinxu Zhao, Siqi Deng, Liman Hou, Juan Song, Ming Wang, Ming Bu

**Affiliations:** College of Pharmacy, Qiqihar Medical University, Qiqihar 161006, China; 17804586082@163.com (S.T.); zyx99813@163.com (Y.Z.); 17735745701@163.com (S.D.); a9059281226a@163.com (L.H.); linchuangyaoli2024@163.com (J.S.)

**Keywords:** lupeol, carbamate, structural modification, hybrid, antitumor

## Abstract

In the following study, a series of new lupeol-3-carbamate derivatives were synthesized, and the structures of all the newly derived compounds were characterized. The new compounds were screened to determine their anti-proliferative activity against human lung cancer cell line A549, human liver cancer cell line HepG2, and human breast cancer cell line MCF-7. Most of the compounds were found to show better anti-proliferative activity in vitro than lupeol. Among them, obvious anti-proliferation activity (IC_50_ = 5.39~9.43 μM) was exhibited by compound **3i** against all three tumor cell lines. In addition, a salt reaction was performed on compound **3k** (IC_50_ = 13.98 μM) and it was observed that the anti-proliferative activity and water solubility of compound **3k·CH_3_I** (IC_50_ = 3.13 μM), were significantly enhanced subsequent to the salt formation process. The preliminary mechanistic studies demonstrated that apoptosis in HepG2 cells was induced by compound **3k·CH_3_I** through the inhibition of the PI3K/AKT/mTOR pathway. In conclusion, a series of new lupeol-3-carbamate derivatives were synthesized via the structural modification of the C-3 site of lupeol, thus laying a theoretical foundation for the design of this new anticancer drug.

## 1. Introduction

Cancer is recognized as one of the most lethal diseases affecting humans, with its annual incidence and mortality rates continuing to be high [1]. At present, a variety of anti-tumor drugs are being developed and applied in clinical practice. Despite clinical use, many anti-tumor drugs are known to have serious side effects, such as those caused by traditional platinum-based anticancer drugs [2,3]. Their broader use is limited due to toxicity and potential drug resistance. Consequently, it is imperative to further develop more effective and low-toxicity anticancer drugs [4].

Natural products have been utilized in medicine for an extensive historical period and continue to be a principal source from which new drugs are derived. Approximately 65% of all new anti-tumor drugs used in clinical practice are natural or nature-based products. Therefore, the search for natural products with novel structures and significant efficacy is one of the most important research directions at present [5,6,7,8]. Pentacyclic triterpenes comprise a wide range of natural products that can be extracted from different fungi, plants, and marine invertebrates. Ursolic acid (UA), glycyrrhizic acid (GA), and oleanolic acid (OA), among other pentacyclic triterpenoids, have garnered substantial interest for their diverse pharmacological profiles. Specifically, potent anticancer, anti-inflammatory, antioxidant, virucidal, and bactericidal properties have been demonstrated by these compounds [9,10,11].

**Figure 1:**
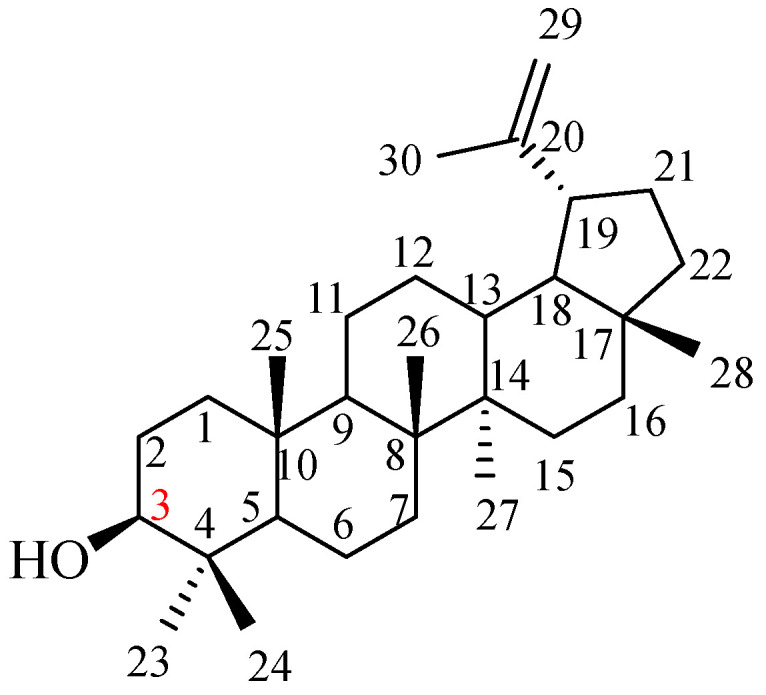
The structure of lupeol (Lup, **1**).

Lupeol (Lup, **1**, Figure 1), a common natural triterpenoid compound, is derived from medicinal plants, as well as edible fruits and vegetables [12]. Recent studies have demonstrated that lupeol possesses anti-inflammatory, anti-tumor, anti-oxidative, and wound healing-promoting properties, with therapeutic effects being exerted on conditions such as arthritis, diabetes, heart disease, nephrotoxic injury, and liver toxic injury [13]. With respect to its anti-neoplastic properties, lupeol has been consistently shown by a wealth of research to exert inhibitory effects on a range of malignant neoplasms, including hepatocellular carcinoma, lung carcinoma, colorectal cancer, prostate cancer, osteosarcoma, and melanoma.

The PI3K/AKT/mTOR pathway has been reported in previous studies to play a pivotal role in the regulation of the cell cycle, autophagy, apoptosis, and other cellular processes across a spectrum of different cell types [14]. The inhibitory effect of silencing the ST3GalIII gene on the proliferation, invasion, and metastasis of human breast cancer cells has been enhanced by lupeol through the suppression of the activation of the PI3K/AKT/mTOR signaling pathway, as reported in recent studies [15]. Lupeol can also induce and enhance the autophagy of cervical cancer cells by inhibiting the phosphorylation of PI3K/AKT/mTOR pathway proteins, reducing their proliferation and invasion activities, and promoting their apoptosis [16]. In addition, the results of other studies have shown that lupeol can inhibit the proliferation of retinoblastoma and promote autophagy and apoptosis, which may also be related to the PI3K/AKT/mTOR pathway [17].

**Figure 2:**
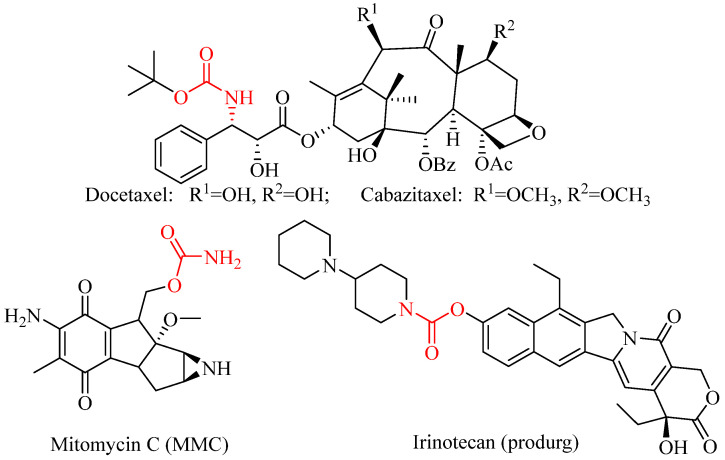
Structure of carbamate compounds.

Carbamate is an organic compound whose amino and carboxyl terminuses are replaced by a variety of alkyl, aryl, or alkyl aryl substituents with different structures, often appearing in the form of -O-CO-NH-linkage, such as docetaxel, mitomycin C, irinotecan, etc. (Figure 2) [18,19]. In recent years, significant interest has been garnered by carbamate derivatives, highlighting their pivotal role in the realm of contemporary drug discovery and medicinal chemistry. The results of recent studies have shown that compounds containing a carbamate group in their molecules can enhance the biological activity of the active pharmacophore of structurally different natural or synthetic compounds [20]. Significant anti-proliferative activities against the human astrocytoma T67 and HeLa cell lines, with IC_50_ values ranging from 0.153 to 0.828 μM, were observed for the pyridine derivatives 8 and 11, which incorporate carbamate group, in studies conducted by Pandolfi F et al. [21]. The activity of butyrylcholinesterase (BuChE) was found to be most potently inhibited by the phenylpiperazine carbamate derivative 16, as reported by Bajda M et al., with an IC_50_ ranging from 1.04 to 3.83 μM [22].

In the past decade, the synthesis of a multitude of carbamate-based compounds has been pursued with vigor, with these compounds emerging as promising candidates in the realm of oncology, either as therapeutic drugs or prodrugs [23]. Certain carbamates have been demonstrated to effectively suppress the proliferation of endothelial cells in vitro and exert a significant inhibitory effect on tumor-induced angiogenesis and tumor growth in murine models in vivo [24]. The results of previous studies show that carbamate derivatives have a good inhibitory effect on tumor cells and show good water solubility [25,26]. Based on the outcomes of the aforementioned studies, a series of lup-3-carbamate derivatives were designed utilizing the biological isostructural substitution method. The biological activities (IC_50_ value) of all derivatives against three human cancer cell lines were evaluated using the MTT method to provide an important reference for the development of new anti-tumor drugs.

**Figure 3:**
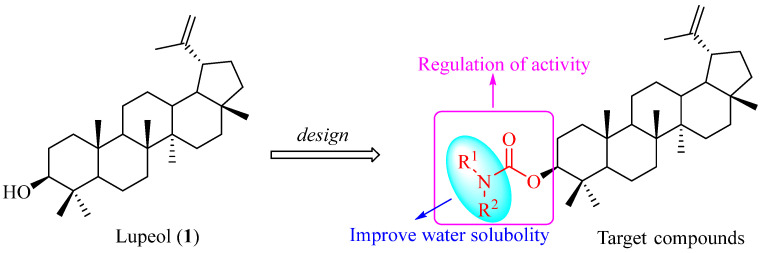
The structure modification strategy of lupeol.

However, it should be noted that lupeol, characterized as a fat-soluble compound with inherently low water solubility, has attributes that may restrict the investigation of its anti-proliferative activity [27,28,29]. Therefore, increasing water solubility and enhancing activity may be important strategies for the structural modification of this compound. To address this issue, in our previous study we found that attaching a structurally simple polar substituent at the C-3 position of **1** can increase its anti-proliferative activity while also increasing solubility. N-heterocyclic rings were found in a variety of bioactive compounds and readily form water-soluble salts with organic or inorganic acids [30]. In light of the above, N-heterocyclic rings at the C-3 position of **1** (Figure 3) were introduced to improve the water solubility and biological activity of the compound. By structurally modifying C-3, potentially important derivatives can be derived, thus promoting its further development as an anti-tumor agent.

## 2. Results and Discussion

### 2.1. Synthesis of Lupeol Derivatives

**Scheme 1:**
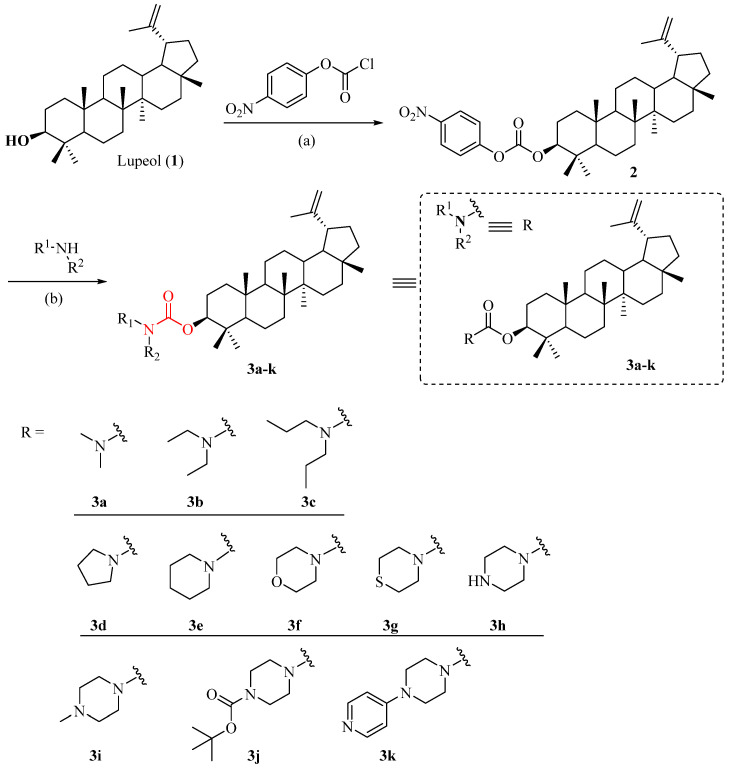
Synthetic pathway to lupeol-3-carbamate derivatives. Reagents and conditions: (**a**) 4-nitrophenyl chloroformate, pyridine, CH_2_Cl_2_, room temperature, 2 h, 80%; (**b**) Et_3_N, CH_2_Cl_2_, various amines, room temperature, 2–5 h, 55%–85%.

**Scheme 2:**
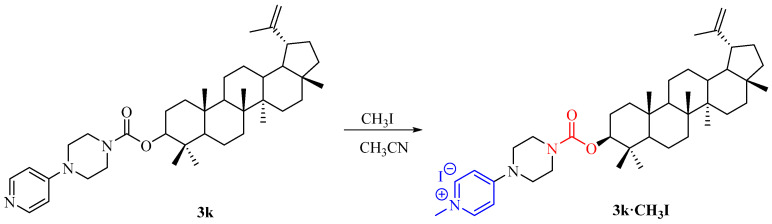
Synthetic pathway to **3k·CH_3_I** derivative. Reagents and conditions: CH_3_I, CH_3_CN, room temperature, 12 h, 73%.

In the present study, a two-step reaction method was designed for the synthesis of lupeol-3-carbamate derivatives (**3a–k**), as shown in Scheme 1. In the first step, lupeol (**1**) was selected as the starting material and was reacted with 4-nitrophenyl chloroformate (PNPCF) in dichloromethane (CH_2_Cl_2_) in the presence of pyridine (Py) as a catalyst, leading to the efficient synthesis of the key intermediate **2**. Subsequently, in the presence of triethylamine (Et_3_N), intermediate **2** was reacted with a series of different amines, and through optimized reaction conditions, we successfully synthesized the target products **3a–k**. Based on Scheme 1, a salt-forming reaction was further designed, employing compound **3k** as the substrate. The reaction with iodomethane (CH_3_I) in acetonitrile (CH_3_CN), conducted under precisely controlled conditions, facilitated the successful synthesis of the target product **3k·CH_3_I**. This synthetic route is detailed in Scheme 2. Unless otherwise specified, all reagents were commercially available and were directly employed in the experiments without further purification. The progress of the reactions was monitored using thin-layer chromatography (TLC) techniques, with observation under ultraviolet (UV) light at 254 nm or staining performed using a 5% sulfuric acid (H_2_SO_4_)/ethanol solution (EtOH) to ensure the smooth progression of the reactions. The chemical structures of all the synthesized compounds were confirmed using analytical techniques such as ^1^H NMR, ^13^C NMR, and HRMS. The experimental section meticulously recorded the physical properties and analytical data of all the synthesized compounds.

### 2.2. Biological Evaluation of Lupeol Derivatives

#### 2.2.1. Lupeol Derivatives Inhibited the Proliferation of Various Human Cancer Cell Lines

The anti-proliferative activities of **3a–k** against human lung cancer cell line (A549), human hepatoma cell line (HepG2), and human breast cancer cell line (MCF-7) were examined. With lupeol and cisplatin as positive controls, the MTT assay was used to evaluate the anti-proliferative activity of all of the synthesized compounds in vitro.

As shown in Table 1, most of the carbamate derivatives (**3a–k**) showed strong inhibitory effects on all three tumor cell lines, and their anti-proliferative activity was higher than that of lupeol. The preliminary structure-activity relationship (SAR) analysis indicated that the presence of nitrogen-containing carbocycle, specifically piperazine, piperidine, and pyridine moieties, significantly enhanced the anti-proliferative activity, as observed with compounds **3e**, **3h**, **3i**, and **3k**, which displayed IC_50_ values less than 20 μM. Notably, the introduction of a methyl group at the para position of the piperazine group (**3i**) resulted in the most pronounced anti-proliferative activity, with an IC_50_ ranging from 5.74 to 9.43 μM. In contrast, compounds substituted with N-methyl, N-ethyl, and five-membered heterocyclic groups (**3a**, **3b**, **3d**) showed relatively lower anti-proliferative activities.

#### 2.2.2. Anti-Proliferative Activity of Selected Compounds in Tumor Cells

**Figure 4:**
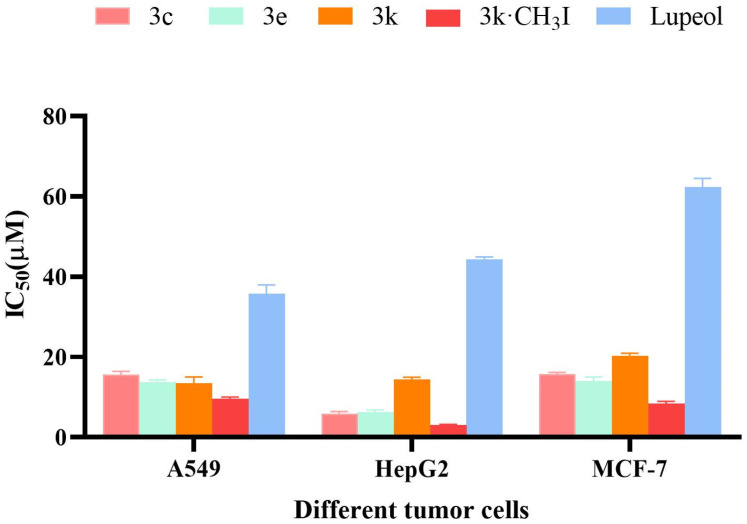
Comparison of the anti-proliferative activity of compounds **3c**, **3e**, **3k**, and **3k·CH_3_I** against different tumor cells.

Due to the unique n-heterocyclic structure of piperazine and pyridinyl, compound **3k** was further reacted with salt, using lupeol as a positive control, and compounds **3c**, **3e**, **3k**, and **3k·CH_3_I** were further tested to determine their anti-tumor activity, as shown in Figure 4. Significant inhibitory effects were observed for the four synthesized compounds on the three tested cell lines, with their anti-proliferative activity being more potent than that of lupeol. Compound **3k·CH_3_I** showed the strongest inhibitory effect on the HepG2 cell line (IC_50_ = 3.13 ± 0.54 μM), with it being about 10 times more active than lupeol. The inhibition of these four compounds on the HepG2 cell line appears to have obvious anti-proliferative activity compared with the MCF-7 and A549 cell lines. The results of our study clearly demonstrate that the anti-proliferative activity of compound **3k** is enhanced subsequent to the salting process. This enhancement suggests that the methyl group is an electron-donating group, leading to an increased electron cloud density on the nitrogen atom after salting. Such an increase in electron density may confer the property of targeting mitochondria, thereby indicating the compound’s potential to be developed as an anticancer agent.

### 2.3. Water Solubility Testing of Compound ***3k·CH_3_I***

**Figure 5:**
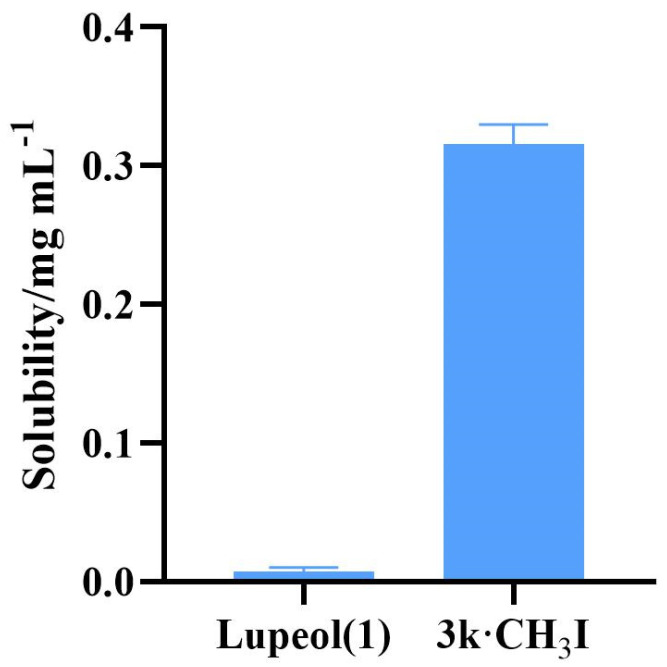
Evaluation of the aqueous solubility of compounds **1** and **3k·CH_3_I**.

The water solubility of compounds **1** and **3k·CH_3_I** was determined, as shown in Figure 5. The concentration gradients of **3k·CH_3_I** were 0.01, 0.03, 0.05, 0.12, 0.20, and 0.35 mg/mL. Compounds **1** and **3k·CH_3_I** were dissolved in 2 mL of distilled water, and distilled water was used as a blank control. The absorbance characteristics of compounds **1** and **3k·CH_3_I** were ascertained utilizing a UV-VIS spectrophotometer at a wavelength of 234 nm. The results showed that compound **3k·CH_3_I** is 60 times more soluble than compound **1**, thus indicating that the water solubility of compound **3k·CH_3_I** significantly improved following salt modification by compounds **1** and **3k**.

### 2.4. Effect of Compound ***3k·CH_3_I*** on the Apoptosis Rate of HepG2 Cells

**Figure 6:**
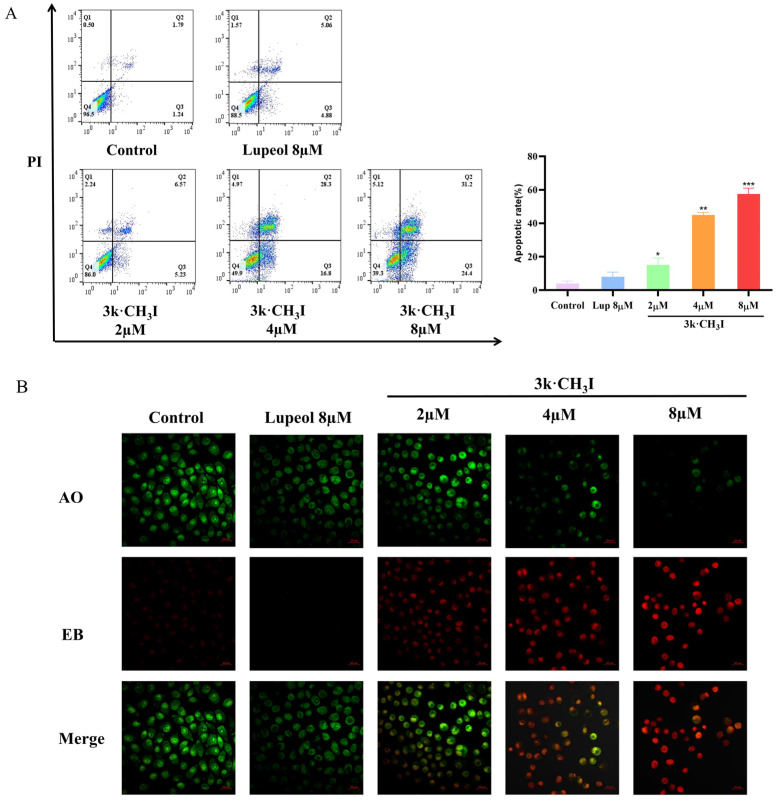
Compound **3k·CH_3_I** affected HepG2 cell growth. (**A**) HepG2 cells were treated with **3k·CH_3_I**, and then, apoptosis was detected using an (AV/PI) assay via flow cytometry. Data are expressed as average ± SD (*n* = 3). * *p* < 0.05, ** *p* < 0.01, and *** *p* < 0.001. (**B**) The effect of **3k·CH_3_I** on the morphological changes in HepG2 cells was determined using the AO/EB method.

The apoptotic morphological alterations induced by compound **3k·CH_3_I** in HepG2 cells were scrutinized after a 48-h treatment using the acridine orange/ethidium bromide (AO/EB) double staining method, as depicted in Figure 6. Normal green fluorescence and no morphological alterations were observed in the control group following AO/EB staining. HepG2 tumor cells treated with compound **3k·CH_3_I** showed bright red fluorescence due to the chromatin concentration and nuclear fragmentation. Concurrently, the apoptosis phenomenon became more obvious with the increase in drug concentration, indicating that compound **3k·CH_3_I** can induce the apoptosis of HepG2 tumor cells in a drug-dependent manner.

Additionally, annexin V/propidium iodide (AV/PI) staining was used to determine the apoptotic rate of HepG2 cells treated with different concentrations of **3k·CH_3_I** via flow cytometry. Following treatment with 0, 2, 4, and 8 μmol/L of compound **3k·CH_3_I** for 48 h, compared with the control group, the proportion of early and late apoptotic cells increased significantly, and the apoptotic rate of HepG2 cells increased with the increase in drug concentration. At concentrations of 4 μmol/L and 8 μmol/L, the total percentage of apoptotic and necrotic cells increased from 3.03% to 45.10% and 55.6% in the normal cell population. The above data indicated that the proliferation of HepG2 cells was inhibited by compound 3k·CH_3_I through the induction of apoptosis.

### 2.5. Effect of Compound ***3k·CH_3_I*** on Reactive Oxygen Species (ROS) in HepG2 Cells

**Figure 7:**
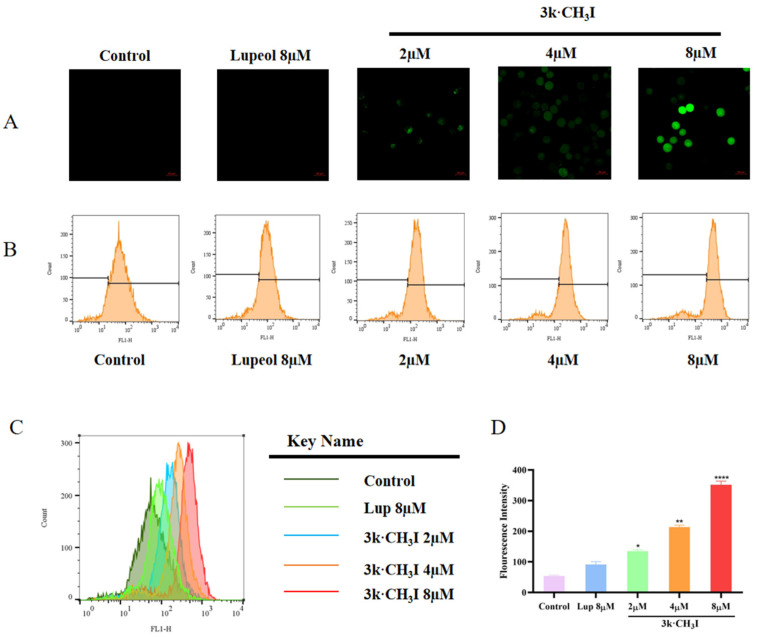
Effect of compound **3k·CH_3_I** on ROS of HepG2 cells. (**A**) Fluorescence microscope view. (**B**) HepG2 cells were treated with **3k·CH_3_I** (0, 2, 4, and 8 μmol/L) for 48 h, stained with DCFH-DA, and then analyzed by flow cytometry. (**C**) Consolidated chart of experimental results. (**D**) Bar chart shows intracellular ROS content at different concentrations. The data were expressed as mean ±SEM, compared with the control group, * *p* < 0.05, ** *p* < 0.01, **** *p* < 0.0001.

As shown in Figure 7, with the increase in drug concentration, the ROS fluorescence in HepG2 cells treated with compound **3k·CH_3_I** was also gradually enhanced. Flow cytometry was additionally used to determine the effect of compound **3k·CH_3_I** on ROS levels in HepG2 cells. After 48 h of drug administration, ROS levels in HepG2 cells treated with compound **3k·CH_3_I** (0, 2, 4, and 8 μmol/L) increased significantly compared with those of the control group. With the increase in drug concentration, these levels increased significantly in a dose-dependent manner. The results of our experiment showed that compound **3k·CH_3_I** significantly increased the production of ROS, which may also be responsible for its induction of apoptosis.

### 2.6. Effect of Compound ***3k·CH_3_I*** on the Mitochondrial Membrane Potential of HepG2 Cells

**Figure 8:**
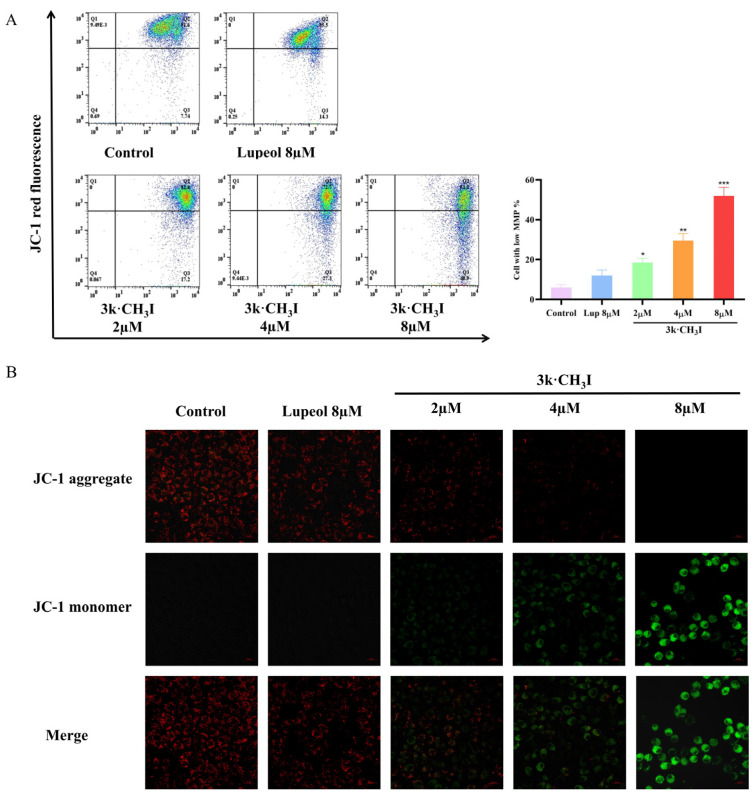
Effect of compound **3k·CH_3_I** on MMP. (**A**) HepG2 cells were treated with **3k·CH_3_I** and then stained with JC-1 dye. Green fluorescence indicates depolarised mitochondria (JC-1 monomer), and red fluorescence indicates hyperpolarised mitochondria (JC-1 aggregation). (**B**) HepG2 cells were treated with **3k·CH_3_I** and then detected via flow cytometry after JC-1 dye staining. Data are expressed as average ± SD (*n* = 3). * *p* < 0.05, ** *p* < 0.01, and *** *p* < 0.001.

In order to explore the effect of compound **3k·CH_3_I** on MMP, a JC-1 staining kit was used to detect the depolarization of the MMP of human hepatocellular carcinoma HepG2 cells treated with compound **3k·CH_3_I**, as shown in Figure 8. Compared with the control group, after HepG2 cells were treated with different concentrations of compound **3k·CH_3_I** for 48 h, the red fluorescence gradually weakened until it eventually disappeared, and the green fluorescence gradually strengthened in HepG2 cells.

Flow cytometry was used to detect the intimal potential of HepG2 cells. It was found that compound **3k·CH_3_I** induced a dose-dependent increase in the MMP depolarization of HepG2 cells at concentrations of 0, 2, 4, and 8 μmol/L. It was found to increase from 7.74% to 17.2%, 27.3%, and 48.9%, respectively. It was concluded that the apoptosis of HepG2 cells was induced by compound **3k·CH_3_I** and was associated with the mitochondrial-mediated endogenous pathway.

### 2.7. Effect of Compound ***3k·CH_3_I*** on the Expression of Related Proteins in HepG2 Cells

**Figure 9:**
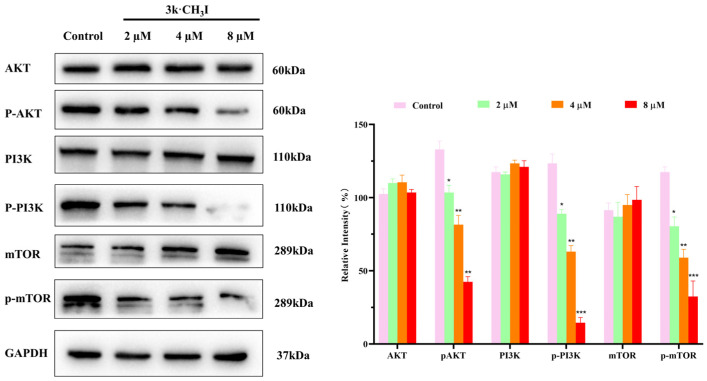
Effects of compound **3k·CH_3_I** on PI3K/AKT/mTOR-related protein expression in HepG2 cells. HepG2 cells were treated with **3k·CH_3_I** (0, 2, 4, and 8 μM) for 48 h. The expression of AKT, P-AKT, P13K, P-P13K, mTOR, p-mTOR, and β-actin was analyzed with Western blotting using the corresponding specific antibodies. Data are expressed as average ± SD (*n* = 3). * *p* < 0.05, ** *p* < 0.01, and *** *p* < 0.001 (Supplementary Materials).

In order to better comprehend the effect of compound **3k·CH_3_I** on the expression of proteins related to the mitochondrial apoptosis pathway, HepG2 cells were treated with **3k·CH_3_I** at different concentrations (0, 2, 4, and 8 μmol/L), and Western blotting was used to determine the expression of related proteins, as shown in Figure 9. Relative to the control group, it was demonstrated that the expression of key regulatory proteins in HepG2 cells was significantly modulated by compound **3k·CH_3_I**. Specifically, a dose-dependent upregulation of the basal forms of AKT, PI3K, and mTOR was induced, while a downregulation of their phosphorylated counterparts, P-AKT, P-PI3K, and P-mTOR, was concurrently triggered. The above results suggested that compound **3k·CH_3_I** can induce the apoptosis of HepG2 cells through an endogenous mitochondrial pathway, and AKT, PI3K, and mTOR may be involved in inducing the apoptosis of human hepatocellular carcinoma HepG2 cells.

## 3. Materials and Methods

### 3.1. Chemistry

All the reagents were purchased from commercial suppliers (Aladdin, Shanghai, China) and utilized without the need for additional purification steps. Silica gel GF254 (Qingdao Ocean Chemical Co., Ltd., Qingdao, China) was employed as the stationary phase for thin-layer chromatography (TLC), with the mobile phase consisting of either petroleum ether and ethyl acetate or dichloromethane and methanol mixtures. The progression of all the chemical reactions was carefully monitored through this technique. Column chromatography was used to purify the intermediates and target derivatives (300–400 mesh silica gel, Hehui, Hangzhou, China). ^1^H NMR and ^13^C NMR spectra were recorded with an Avance DRX400 spectrometer (Bruker, Beijing, China) with TMS as an internal standard, and all the reported chemical shift values are presented in terms of chemical shifts (δ) and expressed in units of parts per million (ppm). Mass spectra were recorded on an Esquire 6000 mass spectrometer (Thermo Fisher Scientific, Waltham, MA, USA).

#### 3.1.1. Synthesis of Lupeol-3-(4-nitrobenzoate) (**2**)

To a solution of lupeol (**1**, 500.0 mg, 1.0 mmol) in anhydrous CH_2_Cl_2_ (20.0 mL), pyridine (278.0 μL, 3.0 mmol, 3.0 eq) and 4-nitrophenyl chloroformate (472.0 mg, 2.0 mmol, 3.0 eq) were added under nitrogen gas. The mixture was stirred at room temperature for 2 h. Following confirmation of completion by TLC, the reaction mixture was subsequently diluted in CH_2_Cl_2_ (50.0 mL) and sequentially extracted with aqueous sodium carbonate and a brine solution. The organic layer was then dewatered using anhydrous Na_2_SO_4_, ensuring the thorough removal of moisture. The solvent was removed under reduced pressure to afford a yellow solid, which was separated by chromatography on silica gel with petroleum ether/ethyl acetate (20:1, *v*/*v*) to obtain the desired intermediate **2**.

White solid (300.0 mg, 77%). ^1^H NMR (600 MHz, CDCl_3_) *δ* 8.27 (d, *J* = 9.2 Hz, 2H, 34-H, 36-H), 7.38 (d, *J* = 9.1 Hz, 2H, 33-H, 37-H), 4.69 (s, 1H, 29-H), 4.57 (s, 1H, 29′-H), 4.44 (dd, *J* = 11.4, 5.1 Hz, 1H, 3-H), 1.04 (s, 3H, CH_3_), 1.01 (dt, *J* = 6.8, 4.0 Hz, 2H), 0.99 (s, 3H, CH_3_), 0.96 (s, 3H, CH_3_), 0.90 (s, 3H, CH_3_), 0.87 (s, 3H, CH_3_), 0.79 (s, 3H, CH_3_). ^13^C NMR (150 MHz, CDCl_3_) *δ* 155.92, 152.57, 151.08, 145.37, 125.39, 121.99, 109.53, 87.78, 55.48, 50.46, 48.40, 48.12, 43.12, 42.98, 40.97, 40.12, 38.43, 38.30, 38.14, 37.20, 35.68, 34.28, 29.95, 28.09, 27.56, 25.18, 23.68, 21.10, 19.42, 18.27, 18.13, 16.52, 16.31, 16.11, 14.66. HRMS (ESI) *m*/*z*: calcd for C_37_H_53_NO_5_ [M + H]^+^: 592.3998, found: 592.4002.

#### 3.1.2. General Procedure for Synthesis of Compounds **3a–k**

Amine (0.3 mmol, 3.0 eq) and triethylamine (Et_3_N, 3.0 eq) in CH_2_Cl_2_ (10.0 mL) were added to intermediate **2** (60.0 mg, 0.1 mmol). After 4 h at room temperature under nitrogen gas, the reaction was completed as verified by TLC control. Then CH_2_Cl_2_ (20.0 mL) and H_2_O (20.0 mL) were added. The organic phase was separated, washed with brine (10 mL), dried over Na_2_SO_4_, and then concentrated on a rotary evaporator to afford the crude product. Purification by column chromatography (CH_2_Cl_2_/methanol, 45:1, *v*/*v*) produced the target compound as a white/yellow solid.

##### Lupeol-3-(dimethyl) Carbamate (**3a**)

Yellow solid (102.5 mg, 66%). ^1^H NMR (600 MHz, CDCl_3_) *δ* 4.61 (s, 1H, 29-H), 4.50 (s, 1H, 29′-H), 4.25 (dd, *J* = 11.8, 4.6 Hz, 1H, 3-H), 2.83 (s, 6H, CH_3_), 1.61 (s, 3H, 30-H), 0.96 (s, 3H, CH_3_), 0.94 (s, 1H, 22′-H), 0.88 (s, 3H, CH_3_), 0.82 (s, 3H, CH_3_), 0.78 (s, 3H, CH_3_), 0.77 (s, 3H, CH_3_), 0.72 (s, 3H, CH_3_); ^13^C NMR (150 MHz, CDCl_3_) *δ* 155.74, 149.98, 108.34, 80.78, 54.33, 49.28, 47.25, 46.99, 41.97, 41.80, 39.82, 38.98, 37.32, 37.06, 37.02, 36.04, 34.55, 33.18, 28.79, 28.67, 27.01, 26.41, 24.07, 23.21, 19.91, 18.25, 17.20, 16.98, 15.72, 15.13, 14.95, 13.52; HRMS (ESI) *m*/*z*: calcd for C_33_H_55_NO_2_Na [M + Na]^+^: 520.4138, found: 520.4130.

##### Lupeol-3-(diethyl) Carbamate (**3b**)

Yellow solid (125.0 mg, 73%). ^1^H NMR (600 MHz, CDCl_3_) *δ* 4.68 (s, 1H, 29-H), 4.56 (s, 1H, 29′-H), 4.34 (dd, *J* = 11.8, 4.6 Hz, 1H, 3-H), 3.26 (m, 4H, 32-H, 33-H), 1.68 (s, 3H, 30-H), 1.11 (t, *J* = 7.1 Hz, 6H, CH_3_), 1.03 (s, 3H, CH_3_), 0.95 (s, 3H, CH_3_), 0.90 (s, 3H, CH_3_), 0.85 (s, 3H, CH_3_), 0.83 (s, 3H, CH_3_), 0.80 (s, 3H, CH_3_); ^13^C NMR (150 MHz, CDCl_3_) *δ* 156.15, 151.14, 109.50, 81.52, 55.55, 50.44, 48.42, 48.15, 43.13, 42.97, 40.98, 40.14, 38.49, 38.21, 38.19, 37.21, 35.71, 34.35, 29.95, 28.20, 27.58, 25.23, 24.27, 21.08, 19.40, 18.37, 18.14, 16.91, 16.29, 16.11, 14.69; HRMS (ESI) *m*/*z*: calcd for C_35_H_59_NO_2_Na [M + Na]^+^: 548.4431, found: 548.4443.

##### Lupeol-3-(dipropyl) Carbamate (**3c**)

White solid (92.5 mg, 56%). ^1^H NMR (600 MHz, CDCl_3_) *δ* 4.61 (s, 1H, 29-H), 4.49 (s, 1H, 29′-H), 4.26 (dd, *J* = 11.8, 4.5 Hz, 1H, 3-H), 3.09 (d, *J* = 20.5 Hz, 4H, 32-H, 33-H), 1.61 (s, 3H, 30-H), 1.48 (d, *J* = 7.7 Hz, 4H, 34-H, 35-H), 0.96 (s, 3H, CH_3_), 0.88 (s, 3H, CH_3_), 0.82–0.78 (m, 12H, CH_3_), 0.77 (s, 3H, CH_3_), 0.72 (s, 3H, CH_3_); ^13^C NMR (150 MHz, CDCl_3_) *δ* 155.42, 149.99, 108.34, 80.40, 54.39, 49.27, 47.26, 46.99, 41.97, 41.81, 39.82, 38.98, 37.32, 37.02, 36.05, 34.55, 33.19, 28.79, 27.03, 26.42, 24.07, 23.06, 19.92, 18.24, 17.20, 16.98, 15.76, 15.11, 14.95, 13.54, 10.26; HRMS (ESI) *m*/*z*: calcd for C_37_H_63_NO_2_Na [M + Na]^+^: 576.4749, found: 576.4756.

##### Lupeol-3-(pyrrolidine-1-carboxylate) (**3d**)

Yellow solid (134.0 mg, 80%). ^1^H NMR (600 MHz, CDCl_3_) *δ* 4.61 (s, 1H, 29-H), 4.50 (s, 1H, 29′-H), 4.26 (dd, *J* = 11.8, 4.6 Hz, 1H, 3-H), 3.32–3.26 (m, 4H, 32-H, 35-H), 1.78 (s, 4H, 33-H, 34-H), 1.61 (s, 3H, 30-H), 0.96 (s, 3H, CH_3_), 0.87 (s, 3H, CH_3_), 0.82 (s, 3H, CH_3_), 0.78 (s, 3H, CH_3_), 0.76 (s, 3H, CH_3_), 0.72 (s, 3H, CH_3_). ^13^C NMR (150 MHz, CDCl_3_) *δ* 154.26, 149.98, 108.34, 80.23, 54.33, 49.29, 47.25, 46.99, 41.97, 41.80, 39.82, 38.98, 37.33, 37.04, 37.02, 36.04, 34.55, 33.20, 28.79, 28.68, 27.05, 26.41, 24.07, 23.31, 19.91, 18.25, 17.22, 16.98, 15.69, 15.15, 14.95, 13.52; HRMS (ESI) *m*/*z*: calcd for C_35_H_57_NO_2_Na [M + Na]^+^: 546.4294, found: 546.4287.

##### Lupeol-3-(piperidine-1-carboxylate) (**3e**)

White solid (126.0 mg, 75%). ^1^H NMR (600 MHz, CDCl_3_) δ 4.61 (s, 1H, 29-H), 4.50 (s, 1H, 29′-H), 4.27 (dd, *J* = 11.8, 4.5 Hz, 1H, 3-H), 3.33 (s, 4H, 32-H, 36-H), 1.61 (s, 3H, 30-H), 1.44 (t, *J* = 6.4 Hz, 6H, 33-H, 34-H, 35-H), 0.96 (s, 3H, CH_3_), 0.88 (s, 3H, CH_3_), 0.81 (s, 3H, CH_3_), 0.78 (s, 3H, CH_3_), 0.76 (s, 3H, CH_3_), 0.72 (s, 3H, CH_3_); ^13^C NMR (150 MHz, CDCl_3_) *δ* 154.64, 149.97, 108.34, 80.53, 54.33, 49.28, 47.25, 46.99, 43.74, 41.97, 41.80, 39.82, 38.98, 37.34, 37.12, 37.02, 36.04, 34.55, 33.18, 28.79, 27.02, 26.41, 24.74, 24.07, 23.49, 23.18, 19.92, 18.25, 17.20, 16.98, 15.80, 15.13, 14.95, 13.52; HRMS (ESI) *m*/*z*: calcd for C_36_H_59_NO_2_Na [M + Na]^+^: 560.4437, found: 560.4443.

##### Lupeol-3-(morpholine-1-carboxylate) (**3f**)

Yellow solid (103.0 mg, 70%). ^1^H NMR (600 MHz, CDCl_3_) *δ* 4.61 (s, 1H, 29-H), 4.50 (s, 1H, 29′-H), 4.31 (dd, *J* = 11.8, 4.5 Hz, 1H, 3-H), 3.58 (s, 4H, 32-H, 35-H), 3.40 (d, *J* = 4.6 Hz, 4H, 33-H, 34-H), 1.61 (s, 3H, 30-H), 0.96 (s, 3H, CH_3_), 0.88 (s, 3H, CH_3_), 0.81 (s, 3H, CH_3_), 0.78 (s, 3H, CH_3_), 0.76 (s, 3H, CH_3_), 0.72 (s, 3H, CH_3_); ^13^C NMR (150 MHz, CDCl_3_) *δ* 155.73, 151.12, 109.51, 82.34, 66.82, 55.48, 50.43, 48.40, 48.14, 43.12, 42.96, 40.96, 40.13, 38.46, 38.26, 38.17, 37.19, 35.70, 34.32, 29.95, 28.20, 27.56, 25.21, 24.28, 21.08, 19.41, 18.34, 18.13, 16.95, 16.27, 16.10, 14.67; HRMS (ESI) *m*/*z*: calcd for C_35_H_57_NO_3_Na [M + Na]^+^: 562.4238, found: 562.4236.

##### Lupeol-3-(thiomorpholine-1-carboxylate) (**3g**)

Yellow solid (152.3 mg, 85%). ^1^H NMR (600 MHz, CDCl_3_) *δ* 4.68 (s, 1H, 29-H), 4.57 (s, 1H, 29′-H), 4.38 (dd, *J* = 11.7, 4.5 Hz, 1H, 3-H), 3.78–3.68 (m, 4H, 32-H, 35-H), 2.59 (d, *J* = 14.8 Hz, 4H, 33-H, 34-H), 1.68 (s, 3H, 30-H), 1.03 (s, 3H, CH_3_), 0.94 (s, 3H, CH_3_), 0.88 (s, 3H, CH_3_), 0.85 (s, 3H, CH_3_), 0.83 (s, 3H, CH_3_), 0.79 (s, 3H, CH_3_); ^13^C NMR (150 MHz, CDCl_3_) *δ* 154.21, 149.97, 108.34, 81.20, 54.31, 49.28, 47.25, 46.98, 41.97, 41.81, 39.81, 38.97, 37.30, 37.11, 37.01, 36.04, 34.54, 33.16, 28.79, 28.68, 27.08, 26.41, 24.05, 23.12, 19.92, 18.25, 17.19, 16.97, 15.85, 15.12, 14.94, 13.51; HRMS (ESI) *m*/*z*: calcd for C_35_H_57_NO_2_NaS [M + Na]^+^: 578.4001, found: 578.4008.

##### Lupeol-3-(piperazine-1-carboxylate) (**3h**)

White solid (122.0 mg, 77%). ^1^H NMR (600 MHz, CDCl_3_) *δ* 4.68 (s, 1H, 29-H), 4.57 (s, 1H, 29′-H), 4.36 (dd, *J* = 11.8, 4.5 Hz, 1H, 3-H), 3.46 (s, 4H, 32-H, 35-H), 2.84 (s, 4H, 33-H, 34-H), 1.68 (s, 3H, 30-H), 1.03 (s, 3H, CH_3_), 0.94 (s, 3H, CH_3_), 0.88 (s, 3H, CH_3_), 0.85 (s, 3H, CH_3_), 0.83 (s, 3H, CH_3_), 0.79 (s, 3H, CH_3_); ^13^C NMR (150 MHz, CDCl_3_) *δ* 155.72, 151.12, 109.50, 82.12, 55.48, 50.42, 48.40, 48.13, 45.94, 43.12, 42.95, 40.96, 40.12, 38.47, 38.26, 38.17, 37.18, 35.70, 34.32, 29.94, 28.19, 27.56, 25.21, 24.30, 21.07, 19.40, 18.34, 18.13, 16.96, 16.27, 16.10, 14.67; HRMS (ESI) *m*/*z*: calcd for C_35_H_59_N_2_O_2_ [M + H]^+^: 539.4580, found: 539.4577.

##### Lupeol-3-((4-methylpiperazine)-1-carboxylate) (**3i**)

Yellow solid (95.7 mg, 55%). ^1^H NMR (600 MHz, CDCl_3_) *δ* 4.62 (s, 1H, 29-H), 4.50 (s, 1H, 29′-H), 4.29 (dd, *J* = 11.8, 4.4 Hz, 1H, 3-H), 3.44 (s, 4H, 32-H, 35-H), 2.35–2.28 (m, 5H, 33-H, 34-H, 19-H), 2.25 (s, 3H, CH_3_), 1.61 (s, 3H, 30-H), 0.96 (s, 3H, CH_3_), 0.87 (s, 3H, CH_3_), 0.81 (s, 3H, CH_3_), 0.78 (s, 3H, CH_3_), 0.76 (s, 3H, CH_3_), 0.72 (s, 3H, CH_3_); ^13^C NMR (150 MHz, CDCl_3_) *δ* 154.47, 149.97, 108.35, 81.04, 54.32, 53.74, 49.27, 47.25, 46.98, 45.07, 42.44, 41.97, 41.80, 39.81, 38.97, 37.31, 37.11, 37.01, 36.03, 34.54, 33.17, 28.79, 27.04, 26.41, 24.05, 23.13, 19.92, 18.25, 17.19, 16.98, 15.80, 15.12, 14.95, 13.52; HRMS (ESI) *m*/*z*: calcd for C_36_H_61_N_2_O_2_ [M + H]^+^: 553.4733, found: 553.4733.

##### Lupeol-3-((4-tert-butyl) piperazine-1)-carbamate (**3j**)

Yellow solid (92.1 mg, 60%). ^1^H NMR (600 MHz, CDCl_3_) *δ* 4.61 (s, 1H, 29-H), 4.50 (s, 1H, 29′-H), 4.30 (dd, *J* = 11.7, 4.4 Hz, 1H, 3-H), 3.38–3.32 (m, 8H, 32-H, 33-H, 34-H, 35-H), 1.61 (s, 3H, 30-H), 1.39 (s, 9H, CH_3_), 0.96 (s, 3H, CH_3_), 0.87 (s, 3H, CH_3_), 0.81 (s, 3H, CH_3_), 0.78 (s, 3H, CH_3_), 0.76 (s, 3H, CH_3_), 0.72 (s, 3H, CH_3_); ^13^C NMR (150 MHz, CDCl_3_) *δ* 154.49, 153.65, 149.97, 108.34, 81.26, 54.32, 49.28, 47.25, 46.98, 41.97, 41.80, 39.81, 38.97, 37.30, 37.11, 37.01, 36.03, 34.54, 33.16, 28.79, 27.36, 27.05, 26.41, 24.05, 23.12, 19.92, 18.25, 17.19, 16.98, 15.82, 15.12, 14.95, 13.51; HRMS (ESI) *m*/*z*: calcd for C_40_H_66_N_2_O_4_Na [M + Na]^+^: 661.4927, found: 661.4920.

##### Lupeol-3-((4-(pyridin-4-yl) piperazine)-1-carboxylate) (**3k**)

Yellow solid (117.0 mg, 75%). ^1^H NMR (600 MHz, CDCl_3_) *δ* 8.22 (d, *J* = 5.7 Hz, 2H, 38-H, 39-H), 6.60 (d, *J* = 5.7 Hz, 2H, 37-H, 40-H), 4.62 (s, 1H, 29-H), 4.50 (s, 1H, 29′-H), 4.33 (dd, *J* = 11.8, 4.4 Hz, 1H, 3-H), 3.56 (s, 4H, 33-H, 34-H), 3.29 (s, 4H, 32-H, 35-H), 1.61 (s, 3H, 30-H), 0.97 (s, 3H, CH_3_), 0.88 (s, 3H, CH_3_), 0.82 (s, 3H, CH_3_), 0.79 (d, *J* = 4.2 Hz, 6H, CH_3_), 0.72 (s, 3H, CH_3_); ^13^C NMR (150 MHz, CDCl_3_) *δ* 154.38, 153.89, 149.96, 148.69, 108.35, 107.43, 81.47, 54.32, 49.28, 47.25, 46.98, 44.83, 41.97, 41.81, 39.81, 38.97, 37.30, 37.12, 37.01, 36.04, 34.54, 33.16, 28.79, 27.09, 26.41, 24.05, 23.14, 19.92, 18.26, 17.19, 16.98, 15.84, 15.13, 14.95, 13.51; HRMS (ESI) *m*/*z*: calcd for C_40_H_62_N_3_O_2_ [M + H]^+^: 616.4845, found: 616.4842.

#### 3.1.3. Synthesis of Compound **3k·CH_3_I**

To a solution of compound **3k** (80.0 mg, 0.1 mmol) in acetonitrile (CH_3_CN, 10.0 mL), CH_3_I (129.0 mg, 1.0 mmol, 7.0 eq) was added, stirred at room temperature for 12 h and filtered. Then the solvent was removed under reduced pressure to afford the crude product. An analytical sample was obtained by recrystallization from acetonitrile.

Yellow solid (112.0 mg, 73%). ^1^H NMR (600 MHz, CDCl_3_) *δ* 8.43 (d, *J* = 6.9 Hz, 2H, 38-H, 39-H), 7.23 (d, *J* = 6.0 Hz, 2H, 37-H, 40-H), 4.70 (s, 1H, 29-H), 4.57 (s, 1H, 29′-H), 4.40 (d, *J* = 11.3 Hz, 1H, 3-H), 4.17 (s, 3H, 41-H), 3.73 (d, *J* = 15.3 Hz, 8H, 32-H, 33-H, 34-H, 35-H), 1.69 (s, 3H, 30-H), 1.04 (s, 3H, CH_3_), 0.95 (s, 3H, CH_3_), 0.89 (s, 3H, CH_3_), 0.87 (s, 3H, CH_3_), 0.86 (s, 3H, CH_3_), 0.79 (s, 3H, CH_3_). ^13^C NMR (150 MHz, CDCl_3_) *δ* 154.38, 153.89, 149.96, 148.69, 108.35, 107.43, 81.47, 54.32, 49.28, 47.25, 46.98, 44.83, 41.97, 41.81, 39.81, 38.97, 37.30, 37.12, 37.01, 36.04, 34.54, 33.16, 28.79, 27.09, 26.41, 24.05, 23.14, 19.92, 18.26, 17.19, 16.98, 15.84, 15.13, 14.95, 13.51; HRMS (ESI) *m*/*z*: calcd for C_41_H_64_IN_3_O_2_ [M + H]^+^: 630.5001, found: 630.4999.

### 3.2. Biological Evaluation

#### 3.2.1. In Vitro Cytotoxicity

The MTT (Beyotime, Shanghai, China) colorimetric method was used to test the antitumor activity of the synthetic products. The test compound and the control compound were prepared into the liquid with a concentration of 1, 5, 10, 20, and 40 μmol/L, respectively. The tested tumor cells (A549, HepG2, MCF-7) (Chinese Academy of Sciences, Shanghai, China) were inoculated into 96 well plates with a density of 2 × 10^4^ cells/mL, and 90 μL PBS was injected into each well. The 96 well plates were cultured overnight in an incubator, and each well was injected with the same concentration of 10 μL of the test compound. After 48 h, 10 μL of MTT solution was injected into each well. After culture for 4 h, the plates were turned upside down and 100 μL DMSO (Kermel, Tianjin, China) was added. The optical density (OD) values of the compound and the control product were determined at 490 nm wavelength with enzyme-labeled apparatus.

#### 3.2.2. Cell Morphology Detection

In this study, human hepatoma HepG2 cells were treated with compound **3k·CH_3_I**, and the tested cells were stained by the AO/EB double staining (KeyGEN BioTECH, Nanjing, China) method to observe the morphological changes of the cells. HepG2 cells of logarithmic growth stage were inoculated into confocal six-well plates, and a volume of 2 mL of the cell suspension, adjusted to a concentration of 1 × 10^6^ cells per milliliter, was introduced into each well. The cells were cultured in an aseptic constant temperature incubator for 24 h, and **3k·CH_3_I** solution with concentrations of 0, 2, 4, and 8 μmol/L was added successively, and the culture continued for 48 h. The cellular specimens were rinsed with phosphate-buffered saline (PBS) on two occasions, after which 500 µL of staining buffer was introduced to the preparation, followed by storage in a light-protected environment for subsequent analysis. AO (5 µL) and EB (5 µL) staining solutions were added to the six-well plates successively, mixed and incubated at 4 °C for 20 min under light protection, then PBS was added and observed under a fluorescence microscope.

#### 3.2.3. Detection of Apoptosis

HepG2 cells with logarithmic growth stage were selected and inoculated into a designated confocal six-well plate with 2 mL cell suspension per well (1 × 10^6^ cells/mL) and cultured in a sterile incubator for 24 h. **3k·CH_3_I** solution with a concentration of 0, 2, 4, and 8 μmol/L was added, and culture continued for 48 h. The cellular preparations were initially rinsed with phosphate-buffered saline (PBS), followed by enzymatic detachment using a 0.25% trypsin solution. Post-collection, the cells underwent centrifugation at 800 revolutions per minute (rpm) for a duration of 5 min to pelletize. The resulting cell pellet was subsequently re-suspended in 1 mL of culture medium designed for cell propagation. The 100 µL cell suspension was transferred into a flow detection tube, with 5 µL AnnexinV added, cultured at room temperature away from light for 15 min, and centrifuged at 800 revolutions per minute (rpm) for a period of 5 min, after which the supernatant containing the staining solution was aspirated. Then, 100 µL of binding buffer for re-suspension and 5 µL of PI dye were added and the solution was mixed well and incubated at low temperature, avoiding light for 15 min. The results were measured by flow cytometry.

#### 3.2.4. Detection of Intracellular ROS

HepG2 cells were inoculated into a six-well plate with 2 mL cell suspension per well at a concentration of 1 × 10^6^ cells/mL overnight in an incubation chamber maintained at 37 °C with a 5% CO_2_ atmosphere. The cells were treated with different concentrations of compound **3k·CH_3_I** (0, 2, 4 and 8 μmol/L) and continued to be cultured for 48 h. The cells underwent a PBS rinse to cleanse them, subsequent digestion with a 0.25% trypsin solution to facilitate detachment, and subsequent centrifugation at 800 revolutions per minute (rpm) for 5 min to sediment the cells. The ensuing cell pellet was then re-suspended in 1 mL of nutrient-rich cell culture medium for further experimental procedures. Then, 5 μmol/L DCFH-DA solution was added to each well and they were incubated for 30 min away from light. After incubation, the cells were digested, the supernatant was centrifuged, and the PBS cell suspension was taken and placed in a flow cytometry to determine the experimental results.

#### 3.2.5. Detection of MMP Depolarization

HepG2 cells with logarithmic growth stage were selected and inoculated into a designated confocal six-well plate with 2 mL cell suspension per well (1 × 10^6^ cells/mL) and cultured in a sterile incubator for 24 h. The cells were treated with different concentrations of **3k·CH_3_I** (0, 2, 4, 8 μmol/L) and continued to be cultured for 48 h. PBS cleans the cells, 0.25% trypsin digests the cells, and collects the cells. After the drug action time was over, 1 mL of pre-configured JC-1 staining solution was added and incubated for 30 min. After JC-1 incubation, the cells were observed by fluorescence microscope, the supernatant was separated, and the PBS cell suspension was taken and the experimental results were determined by flow cytometry.

#### 3.2.6. Protein Immunoblot Assay

HepG2 cells with logarithmic growth stage were selected to make a single-cell suspension and inoculated into a culture bottle with a cell density of 1 × 10^6^ cells/mL. The cells were treated with different concentrations of compound **3k·CH_3_I** (0, 2, 4 and 8 μmol/L) and cultured for 48 h. A mixture of 500 μL of phenylmethylsulfonyl fluoride (PMSF) and radio immunoprecipitation assay (RIPA) lysate was added to each well, and the protein content was determined using the bicinchoninic acid (BCA) assay and denatured at high temperature 30 min later. The protein samples were separated by polyacrylamide gel electrophoresis (SDS-PAGE) and transferred to a polyvinylidene fluoride (PVDF) membrane. The membrane was enclosed in 5% skim milk for 2 h, then specific antibodies of different proteins were added, incubated at 4 °C overnight, and secondary antibodies were added for 2 h. Finally, the gel imager was used for detection. In each experiment, β-actin was used as the internal parameter correction. The gray-scale of the bands was analyzed by gel imaging image analysis software and the relative protein content was calculated.

## 4. Conclusions

In conclusion, a series of new lup-3-carbamate derivatives were synthesized from lupeol through an efficient two-step process. All of the compounds were characterized using ^1^H NMR, ^13^C NMR, and HRMS. The results of a bioactivity assay showed that strong anti-proliferative activities were exhibited by the majority of the synthesized compounds against three tumor cell lines in vitro. It is worth noting that compound **3k·CH_3_I** showed a strong inhibitory effect in the human liver cancer cell line (HepG2), with an IC_50_ value of 3.13 μM. In addition, the solubility of compound **3k·CH_3_I** was found to be 60 times that of lupeol. In this study, the antitumor mechanism of action of compound **3k·CH_3_I** was preliminarily investigated. The experimental results indicated that this compound significantly reduced the mitochondrial membrane potential in HepG2 cells, a change that was closely associated with a marked increase in the levels of ROS. Furthermore, apoptosis in HepG2 cells was promoted by the compound through the modulation of the PI3K/AKT/mTOR signaling pathway. In summary, lupeol-3-carbamate derivatives have been demonstrated to exert a pronounced influence on anti-tumor activity, particularly at the C-3 position, thus providing a substantial foundation for the advancement of novel triterpenoid-based antineoplastic agents. In addition, the solubility and activity of compound **3k·CH_3_I** were significantly improved, thus providing new prospects for the study of the salt-forming reaction of these types of compounds in the future.

## Figures and Tables

**Table 1:** Anti-proliferative activities of compounds **3a–k**.

Compound	IC_50_ (μM) ^a^		
	A549	HepG2	MCF-7
**3a**	14.04 ± 1.03	11.11 ± 1.45	10.18 ± 1.24
**3b**	14.04 ± 1.25	13.83 ± 1.23	12.68 ± 1.43
**3c**	15.51 ± 1.20	5.39 ± 0.77	15.29 ± 1.11
**3d**	11.34 ± 1.14	17.68 ± 1.35	12.39 ± 1.55
**3e**	13.18 ± 1.56	6.91 ± 1.60	13.99 ± 1.58
**3f**	6.76 ± 0.96	10.82 ± 1.52	11.26 ± 1.21
**3g**	8.19 ± 1.02	10.12 ± 1.78	7.77 ± 1.09
**3h**	12.03 ± 1.47	11.19 ± 1.45	9.78 ± 1.20
**3i**	5.74 ± 0.83	9.43 ± 1.21	8.20 ± 1.46
**3j**	9.84 ± 1.21	14.46 ± 1.57	18.36 ± 1.78
**3k**	12.12 ± 1.35	13.98 ± 1.65	19.88 ± 1.82
**Lupeol**	35.86 ± 1.58	43.62 ± 1.37	62.03 ± 1.79
**Cisplatin**	4.65 ± 0.52	4.05 ± 0.45	5.27 ± 0.68

^a^ IC_50_ was calculated from three independent experimental measurements using an MTT assay after 48 h of treatment.

## Data Availability

The data will be provided upon request.

## Supplementary Materials

The following supporting information can be downloaded at: https://www.mdpi.com/article/10.3390/molecules29173990/s1, Nuclear magnetic and high resolution mass spectra of compounds.
