# Supplementary figures and images for: Lupeol-3-carbamate Derivatives: Synthesis and Biological Evaluation as Potential Antitumor Agents
Source: Molecules. 2024 Aug 23;29(17):3990. doi: 10.3390/molecules29173990 (PMC11396318; doi:10.3390/molecules29173990)

# AKT 60KDa

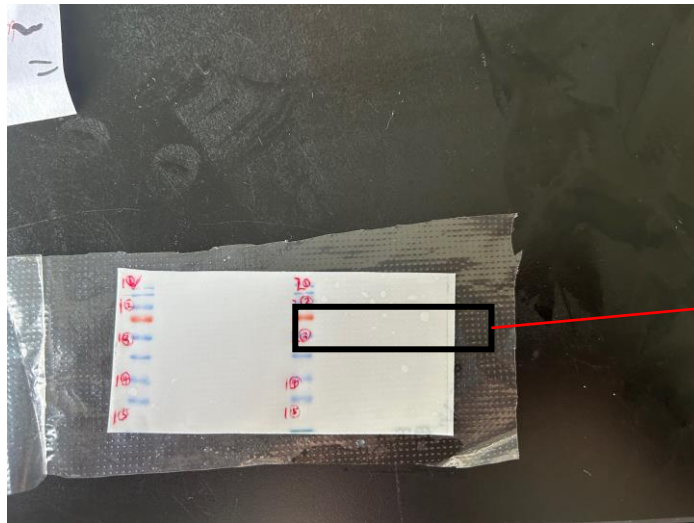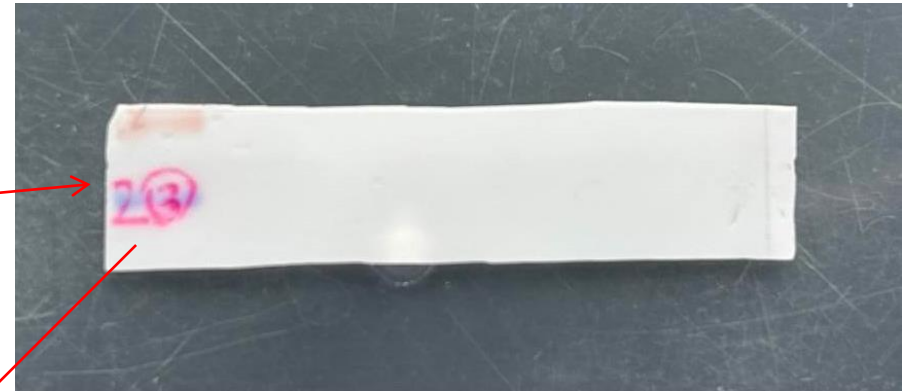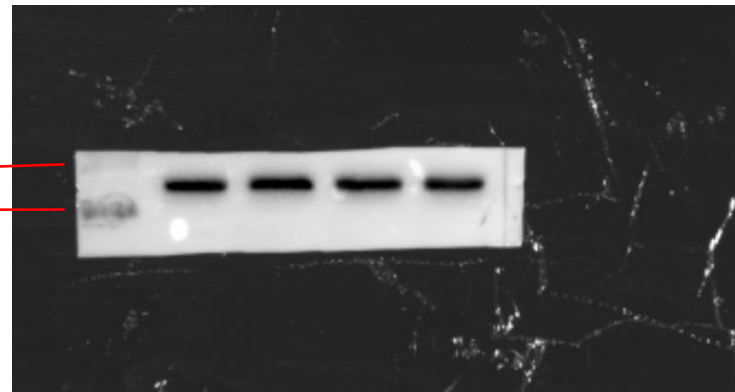

70kda  
55kda

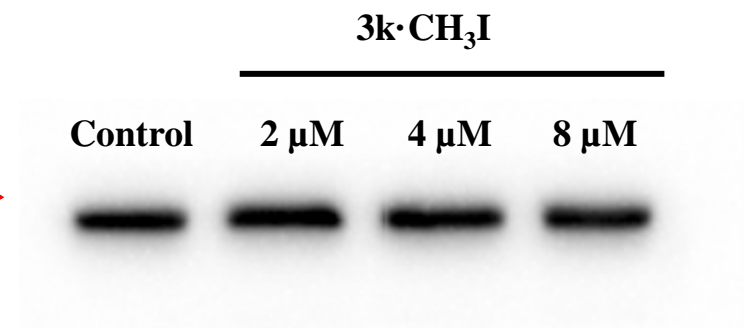

# P-AKT 60KDa

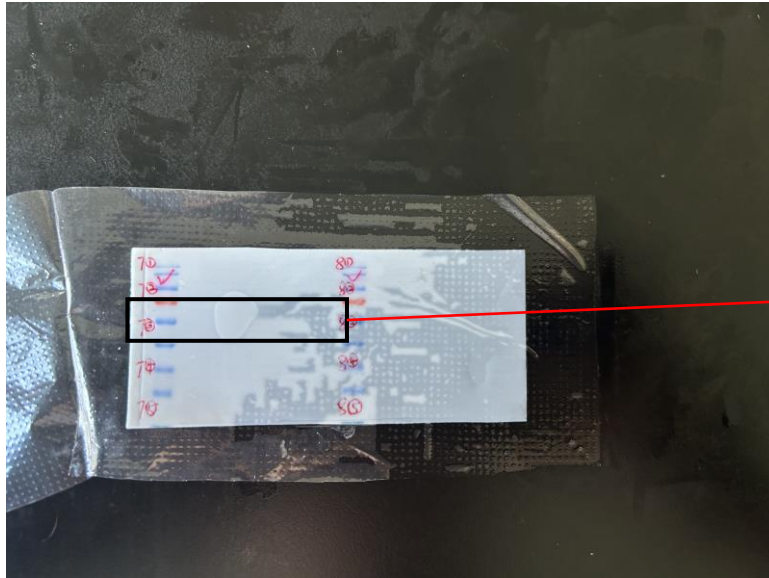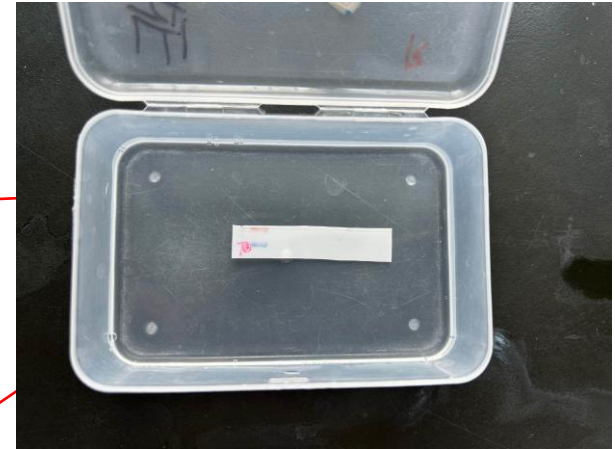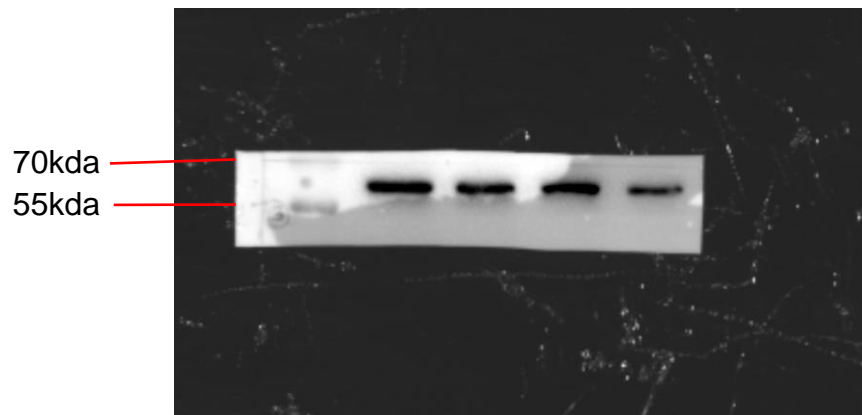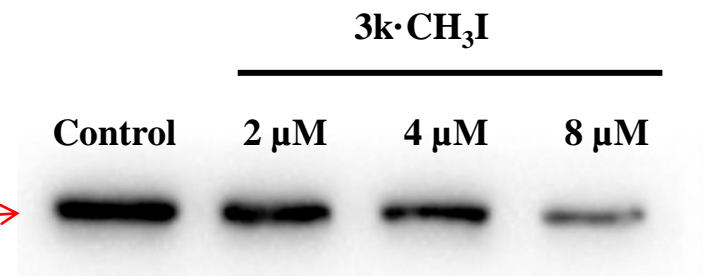

# PI3K 110KDa

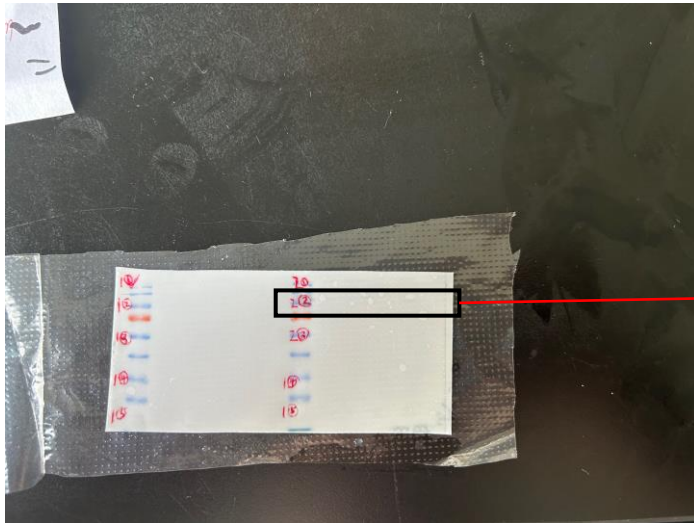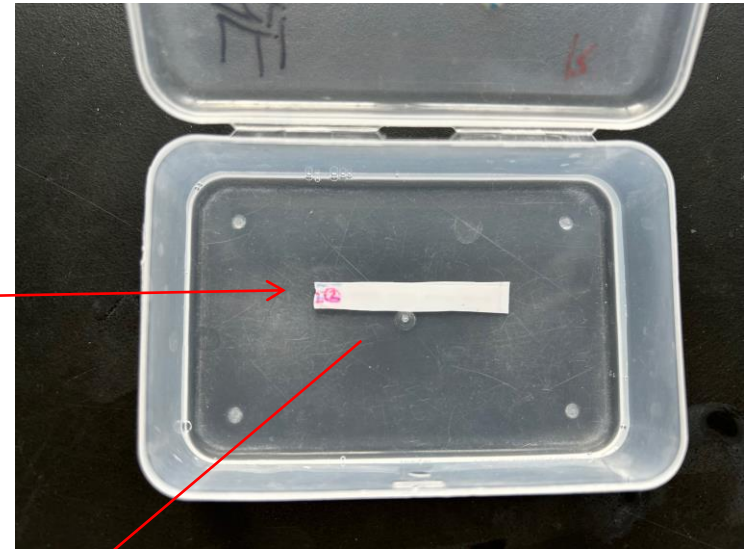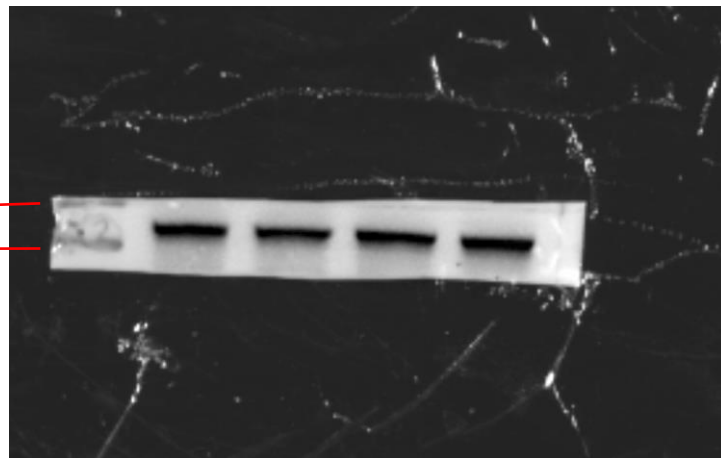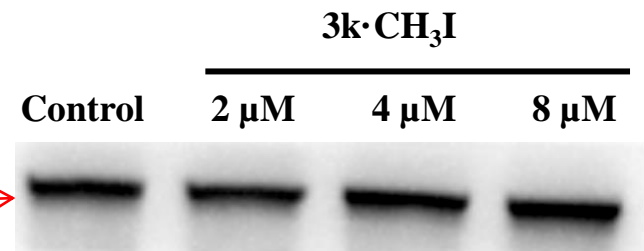

# P-PI3K 110KDa

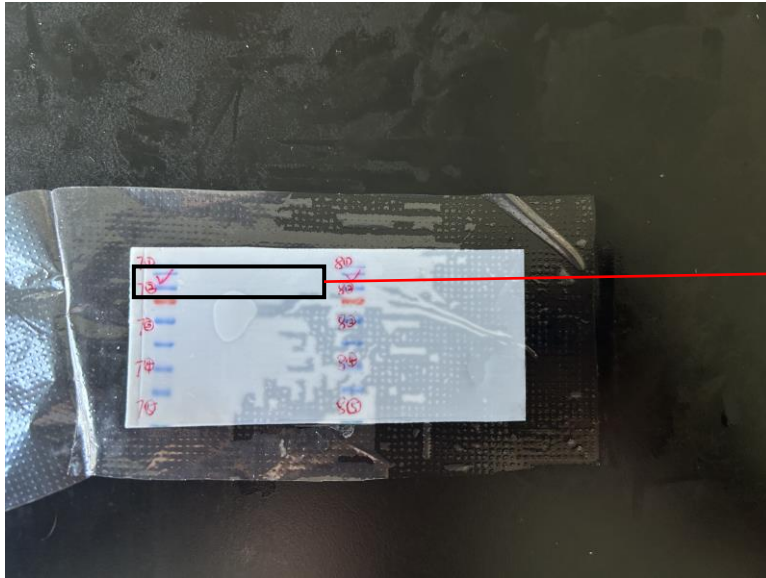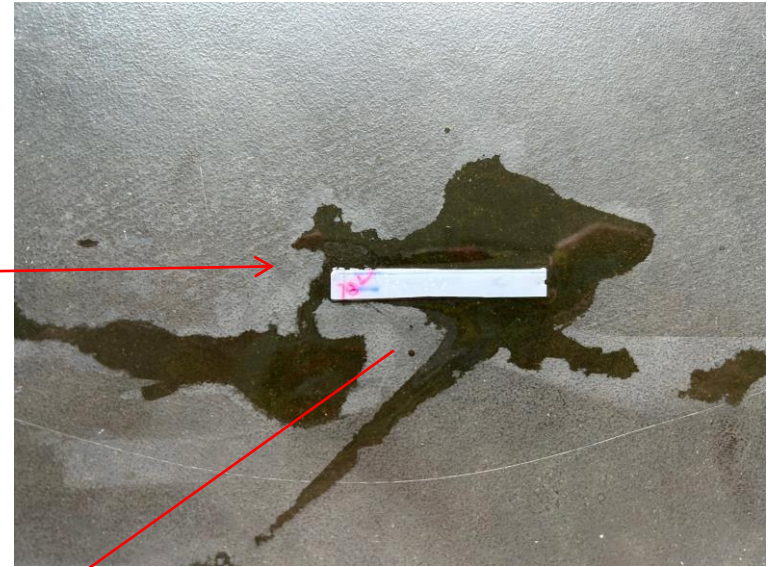

130kda  
100kda

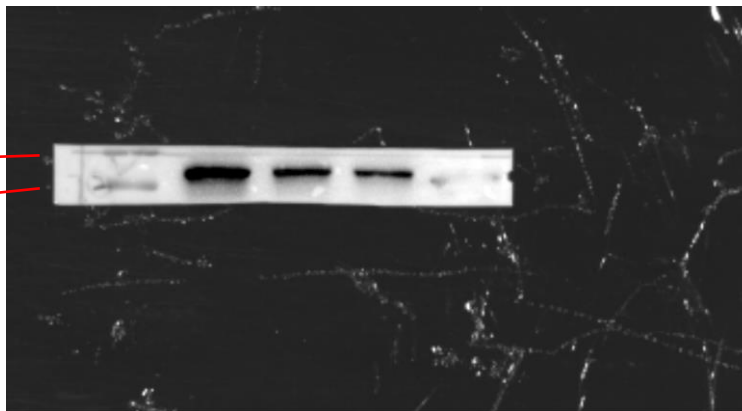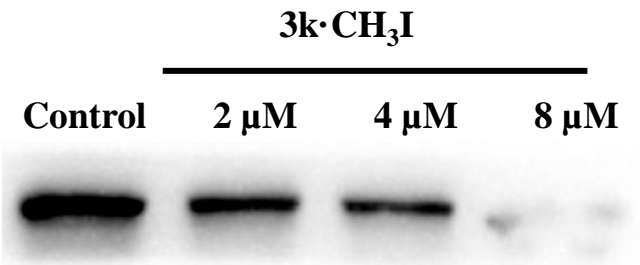

mTOR 289KDa

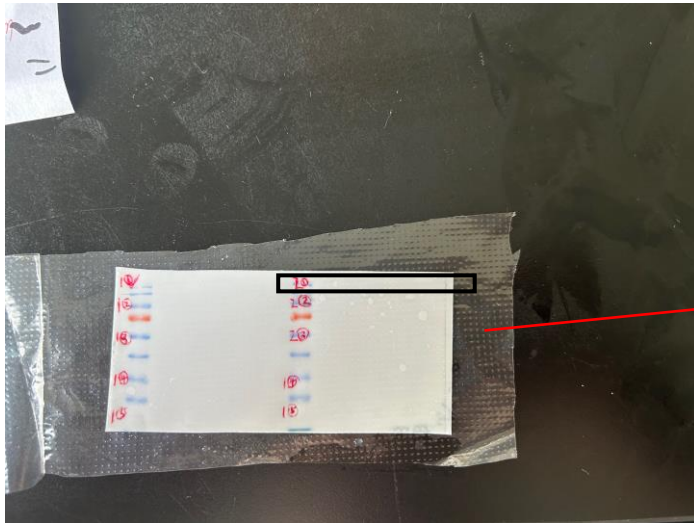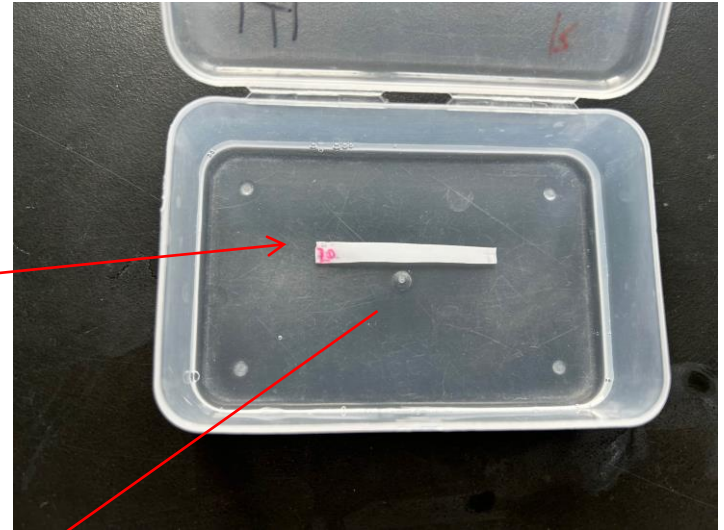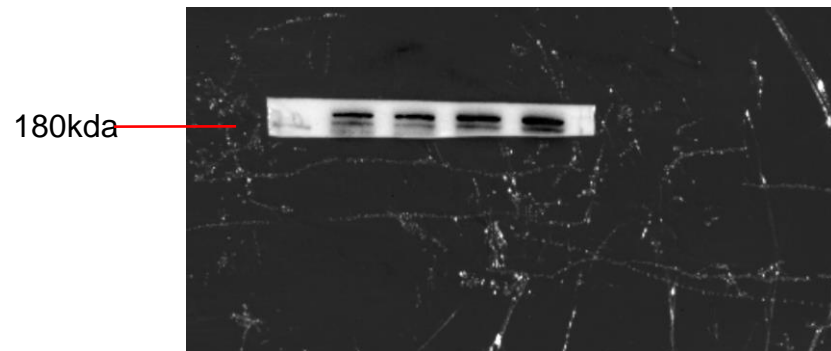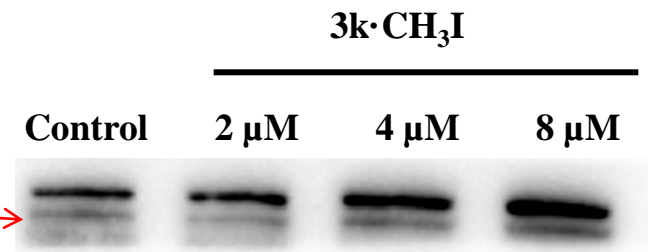

# P-mTOR 289KDa

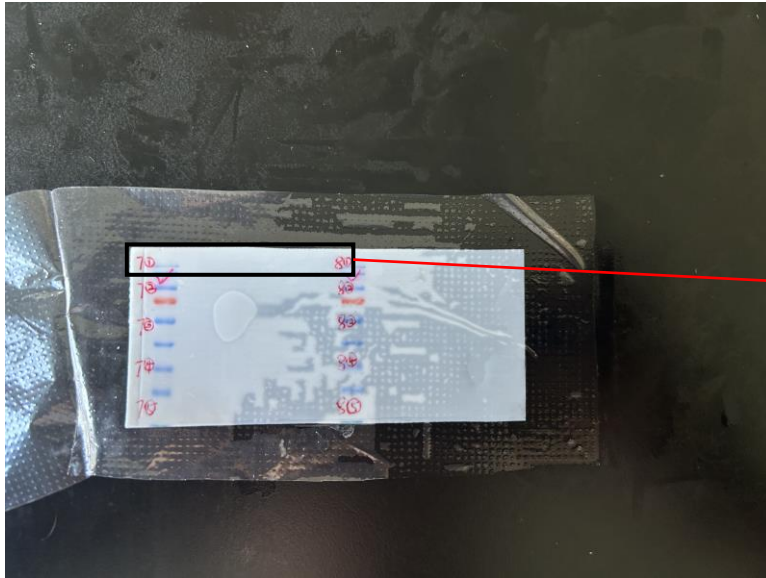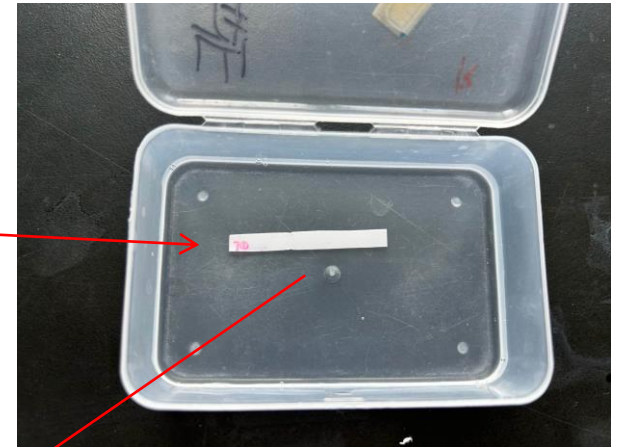

180kda

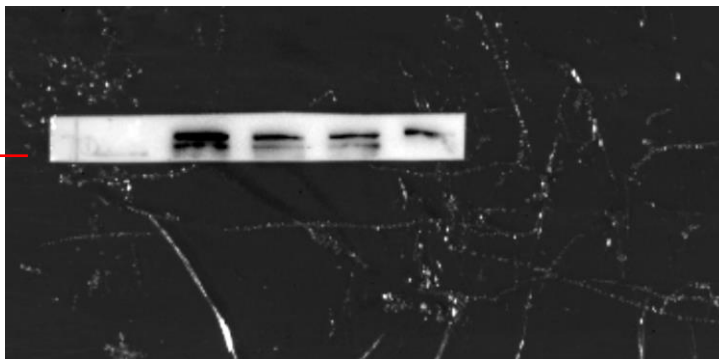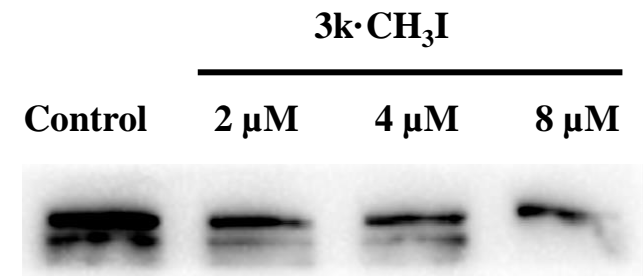

# GAPDH 37KDa

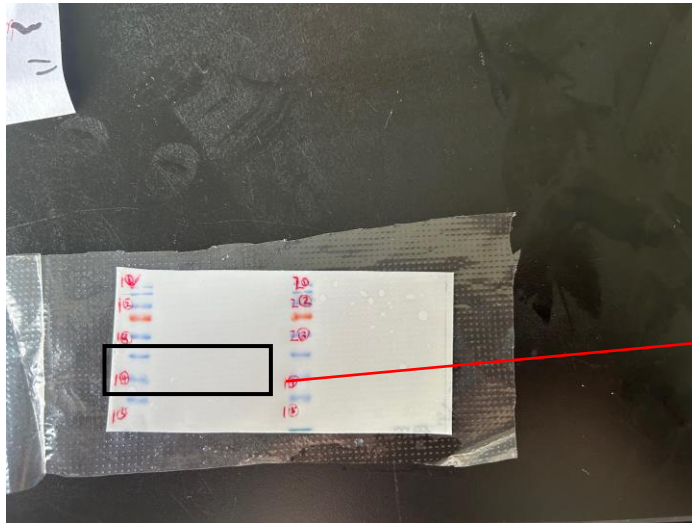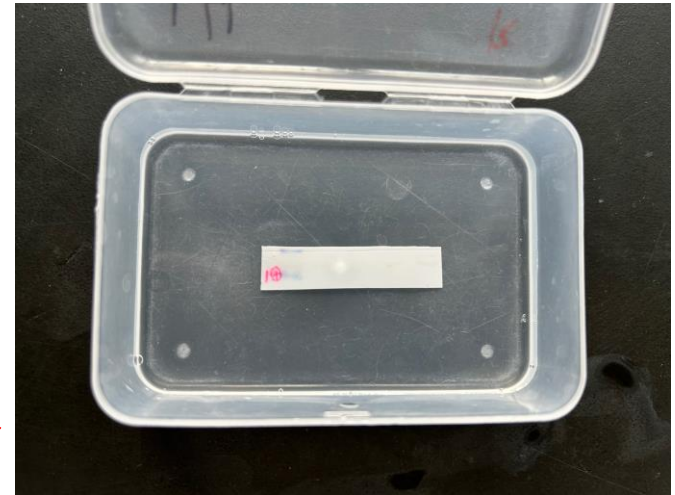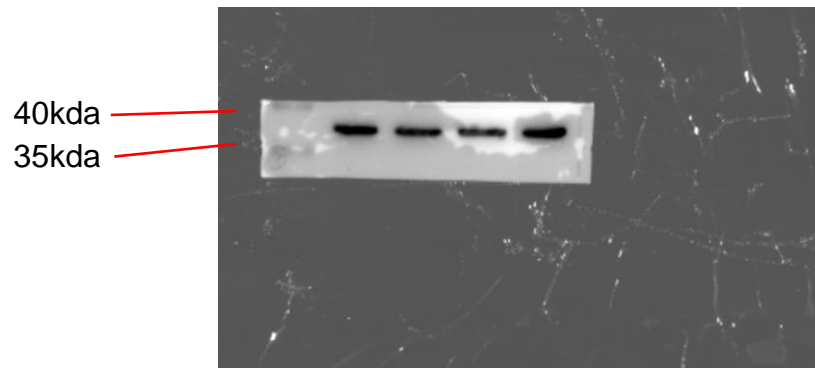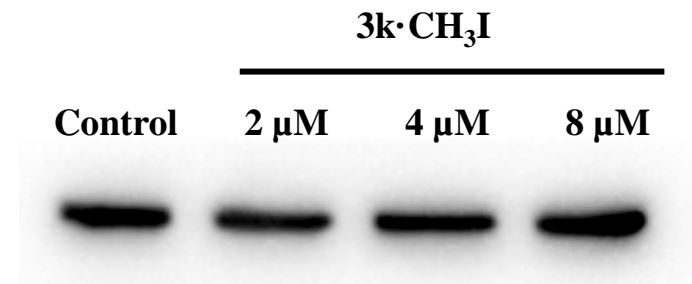

Supplement: Supplementary file 1 [file molecules-29-03990-s001.zip › molecules-3131563-supplementary.pdf]
